# Serum metabolic profile and metabolome genome-wide association study in chicken

**DOI:** 10.1186/s40104-023-00868-7

**Published:** 2023-05-04

**Authors:** Jing Tian, Xiaoning Zhu, Hanyu Wu, Yuzhe Wang, Xiaoxiang Hu

**Affiliations:** 1 State Key Laboratory of Agrobiotechnology, College of Biological Sciences, China Agricultural University, Beijing, 100193 China; 2 National Research Facility for Phenotypic and Genotypic Analysis of Model Animals (Beijing), China Agricultural University, Beijing, 100193 China

**Keywords:** Chicken, Metabolic traits, mGWAS, Serum

## Abstract

**Background:**

Chickens provide globally important livestock products. Understanding the genetic and molecular mechanisms underpinning chicken economic traits is crucial for improving their selective breeding. Influenced by a combination of genetic and environmental factors, metabolites are the ultimate expression of physiological processes and can provide key insights into livestock economic traits. However, the serum metabolite profile and genetic architecture of the metabolome in chickens have not been well studied.

**Results:**

Here, comprehensive metabolome detection was performed using non-targeted LC–MS/MS on serum from a chicken advanced intercross line (AIL). In total, 7,191 metabolites were used to construct a chicken serum metabolomics dataset and to comprehensively characterize the serum metabolism of the chicken AIL population. Regulatory loci affecting metabolites were identified in a metabolome genome-wide association study (mGWAS). There were 10,061 significant SNPs associated with 253 metabolites that were widely distributed across the entire chicken genome. Many functional genes affect metabolite synthesis, metabolism, and regulation. We highlight the key roles of *TDH* and *AASS* in amino acids, and *ABCB1* and *CD36* in lipids.

**Conclusions:**

We constructed a chicken serum metabolite dataset containing 7,191 metabolites to provide a reference for future chicken metabolome characterization work.

Meanwhile, we used mGWAS to analyze the genetic basis of chicken metabolic traits and metabolites and to improve chicken breeding.

**Supplementary Information:**

The online version contains supplementary material available at 10.1186/s40104-023-00868-7.

## Background

Metabolomics studies small molecules (< 1,000 Da) present in biological samples [1]. Essential for growth and health in organisms, metabolites are the final products of gene transcription and protein expression, and are affected by both internal and external factors [2, 3]. Generally regarded as a bridge between genes and phenotypes [4, 5], the combination of metabolomics with genomics and transcriptomics has proven to be powerful in analyzing metabolic diversity and pathways [6, 7]. For example, metabolome genome-wide association studies (mGWAS) in crops such as tomato, corn, and wheat have revealed that many metabolite-associated loci control the effects of fruit color, crop yield, and enzyme activity on the metabolism of specific substances [4, 8, 9]. Metabolite GWAS has found effective therapeutic targets for metabolic diseases, such as human kidney disease and type 2 diabetes [10].

Growth in chickens is determined by quantitative traits regulated by multiple genes [11–14]. Traditional genome-wide association studies (GWAS) can identify SNPs associated with phenotypes but have limited ability to analyze the mechanisms underlying these phenotypes [15, 16]. Investigating metabolic phenotypes (metabotypes)—which are determined by the interaction of genetics and environment—instead of traditional complex phenotypes may help to solve this problem [17]. Currently, the mGWAS approach in livestock has been limited to small-sample comparisons so there is a need to characterize metabotypes in population samples.

Blood contains a variety of substances required for maintaining normal physiological functioning; this makes it a powerful tool for assessing the nutritional and health status of humans and animals. In agricultural animal studies, the association of serum and plasma metabolites with disease [18], meat quality traits [19], feed intake [20] and growth traits [21] has been reported. Serum and plasma are now commonly used as biological samples in metabolomic studies because of their easy accessibility and ability to reflect the overall metabolic characteristics of an organism [22, 23].

In this study, we aim to examine serum metabolome of chickens using a non-targeted metabolomics approach and construct a metabolite dataset for chickens. At the same time, the metabolic phenotype was used for genome-wide association analysis to analyze its genetic model and identify genes related to metabolite synthesis and metabolic pathways. This could greatly improve our understanding of chicken serum metabolic profiles and metabolic phenotypes, providing a strong foundation for future studies on the mechanisms and localization of chicken economic traits.

## Materials and methods

### Advanced intercross line

We created the AIL in this study by crossing individuals from two distinct chicken lines, namely a quality chicken Line A03 (HQLA) and a native Chinese breed Huiyang Bearded chicken (HB). Detailed feeding patterns, as well as F0–F2 mating schemes, were described in a previously published article [24]. Later, AIL generations (F3 to F16) were established from birds in the F2 population and reproduced using random mating [13, 25].

### Serum sample collection and processing

Metabolomics was used to study the serum of 508 12-week-old chickens (266 hens and 242 cocks) of the F16 generation. A serum sample was obtained by centrifugation at 2,000 × *g* for 10 min after blood samples from chickens were left at room temperature. These samples were frozen with liquid nitrogen and then stored at −80 ℃ for later analyses.

All frozen serum samples were initially thawed on ice and vortexed, and 400 μL cold methanol/acetonitrile mixed extract (1:1, v:v) was used for metabolome extraction and protein removal for each 100 μL serum [26]. The supernatant (200 μL) was rotated and dried for analysis. Dried supernatants were then reconstituted in 50 µL of water with 50% methanol (T3 sample) and 94% acetonitrile (Amide sample).

### Metabolite analysis by LC–MS/MS

A Vanquish UHPLC system was coupled to a Q-Exactive HF-X Hybrid Quadrupole-Orbitrap Mass spectrometer (Thermo Fisher Scientific, Waltham, Massachusetts, USA) for non-targeted metabonomics detection. Chromatographic separation was performed using a reverse-phase ACQUITY UPLC HSS T3 column (100 Å, 1.8 μm, 100 mm × 2.1 mm, Waters, Milford, Massachusetts, USA) at 40 ℃ with mobile phases of water containing 0.1% formic acid (A1) and methanol (B1) and HILIC-phase ACQUITY UPLC BEH Amide column (130 Å, 1.7 μm, 100 mm × 2.1 mm, Waters) at 40 °C with mobile phases of 50% acetonitrile with 10 mmol/L ammonium acetate (A2) and 95% acetonitrile with 10 mmol/L ammonium acetate (B2), pH 9. One microliter of pretreated sample was injected. The T3 flow rate was 0.2 mL/min, and gradient elution was performed as follows: the system was equilibrated with A1 for 7 min followed by linear increases to 98% B1 over 26 min and maintained at 98% for 5 min. The amide flow rate was 0.2 mL/min, and gradient elution was performed as follows: the system was equilibrated with 98% B2 for 9.9 min followed by linear reduced to 2% B2 over 20 min and maintained at 2% for 2 min. The mass spectrometer was operated in positive ion mode with a spray voltage of 3,500 V, a capillary temperature of 350 °C, a sheath gas flow rate of 30 arb, an auxiliary gas flow rate of 11 arb, and a probe heater temperature of 220 °C.

Samples were scanned using the full-MS mode, with the resolution of the full scan set at 120,000 and a scan range of *m/z* = 70–1,050. To collect sufficient MS/MS information for metabolite identification, Quality Control (QC) samples underwent segmented secondary scans. These consisted of four sections: *m/z* = 70–160, *m/z* = 150–260, *m/z* = 250–410 and *m/z *= 400–1,050. Using full MS-dd MS2 scan mode, full MS resolution, 60,000; dd-MS2 resolution, 15,000; top N, 15; isolation window, 1 *m/z*; stepped NCE at 20, 30, and 40. The final four-mode data were obtained using T3-pos, T3-neg, Amide-pos and Amide-neg representations.

### Metabolite identification and classification

XCMS is a software based on R language which is often used for LC–MS data pre-processing analysis [27] and in our study it was used for peak extraction, peak alignment, etc. The parameters were the maximum allowable deviation in *m/z* for continuous scanning: ppm = 20, the range of peak widths: peakwidth = c (5, 34), the the signal-to-noise ratio threshold: snthresh = 4, the prefiltering step in the first step: prefilter = c (3, 15,000), the inclusiveness of the grouping: bw1 = 15 and bw2 = 5 to obtain the initial MS feature. One-MAP (One-step Metabolomics: A Smart Cloud Platform for Metabolites Identification and Biomarkers Discovery, www.5omics.com) was used to identify the metabolites of segmented secondary QC sample data. Three classes of databases of One-MAP were used for metabolite identification: a standard database, which structurally identified metabolites through direct comparison of their chromatographic and fragmentation behavior with 1,500 standards [28], a KEGG database, and an integrated database.

We used ClassyFire (https://cfb.fiehnlab.ucdavis.edu/), an online metabolite classification software, to classify the identified metabolites into substances [29]. We also combined HMDB and KEGG databases to classify metabolites by internal and external sources: Among the small-molecule metabolites, those clearly attributed to plant and drug sources were classified as exogenous metabolites, while the rest were considered endogenous metabolites (vitamins and hormones require specific analysis), and all conventional lipids were considered endogenous metabolites.

### Statistical analysis

The metabolite data were log10-transformed to improve normality for statistical analysis. Coefficient of variation (CV) values were calculated for each metabolite and expressed as S/A, where S and A represent the standard deviation and mean of the metabolites in the population, respectively. Pearson's correlations between metabolites and statistical significance were estimated using R (http://www.r-project.org/). Metabolite pathway analysis was performed using the online metabolite pathway enrichment software MetaboAnalyst 5.0 [30]. Gene function enrichment was performed using the Metascape software [31].

### Genotypic information

DNA was extracted from blood samples using the DNeasy Blood & Tissue Kit (Qiagen 69506, Hilden, GER), evaluated using a NanoDrop spectrophotometer (Thermo Fisher Scientific), and examined on 1% agarose gels. All samples were quantified using a Qubit 2.0 fluorometer (Invitrogen, Carlsbad, California, USA) and then diluted to 40 ng/mL in a 96-well plate. Libraries were constructed using the Tn5 method, and final libraries were sequenced on two lanes of an MGISEQ-2000 (MGI, Shenzhen, Guangzhou, CHN) to generate 2 × 100 bp double-end reads or on one lane of a BGISEQ-500 (MGI, Shenzhen, Guangzhou, CHN) to generate 2 × 100 bp double-end reads [32].

In summary, low-coverage sequencing data from more than 1,000 samples were mapped to the GRCg6a reference genome and the BaseVar + STITCH pipeline was used to impute SNPs [32]. A subset of 962,660 SNPs that tagged all other SNPs with MAF > 5% at LD *r*^2^ > 0.95 were used for subsequent analysis.

### mGWAS and heritability estimation

To integrate genomic and metabolomic data, metabolome genome-wide association studies (mGWAS) were conducted in which each metabolite (*n* = 2,935) was considered a phenotype and examined for its association with each SNP (960 K). The mixed linear model approach was used for genome-wide association analysis based on marker SNPs, as implemented in the GCTA (1.93.2) package [33]. We estimated SNP heritability using the GREML module in the software package GCTA (1.93.2) metabolites heritability estimation. Heritability was estimated using a mixed model as follows:

$${\varvec{y}}\boldsymbol{ }={\varvec{Xb}} +{\varvec{Wu}}\boldsymbol{ }+\boldsymbol{ }{\varvec{e}}$$

with var (***y***) = ***WW****'σ*_*u*_^*2*^ + ***I****σ*_*e*_^*2*^, where ***y*** is the vector of the metabolite phenotypes, ***b*** is a vector of the fixed effects (sex and batch), with its incidence matrix ***X***, ***u*** is the vector of additive values based on the genotype data, and ***e*** is a random residual error. ***W*** is a genomic additive relationship matrix, *σ*_*u*_^*2*^ is the additive variance, and *σ*_*e*_^*2*^ is the residual variance. Variance components were estimated by genome-based restricted maximum likelihood (GREML) using the reml program in GCTA [34].

### SNP annotation and candidate genes

We first used SnpEff software [35] to annotate functional genes for 10,061 SNPs that reached the significance threshold. The genes obtained from mGWAS analysis of the same metabolite species were enriched for functions using Metascape software [31] to screen candidate genes related to the regulation, synthesis, and metabolism of the species, and the SNPs annotated to the respective gene were considered key SNPs.

## Results

### Serum metabolic profiling and non-targeted metabolite dataset

**Fig. 1:**
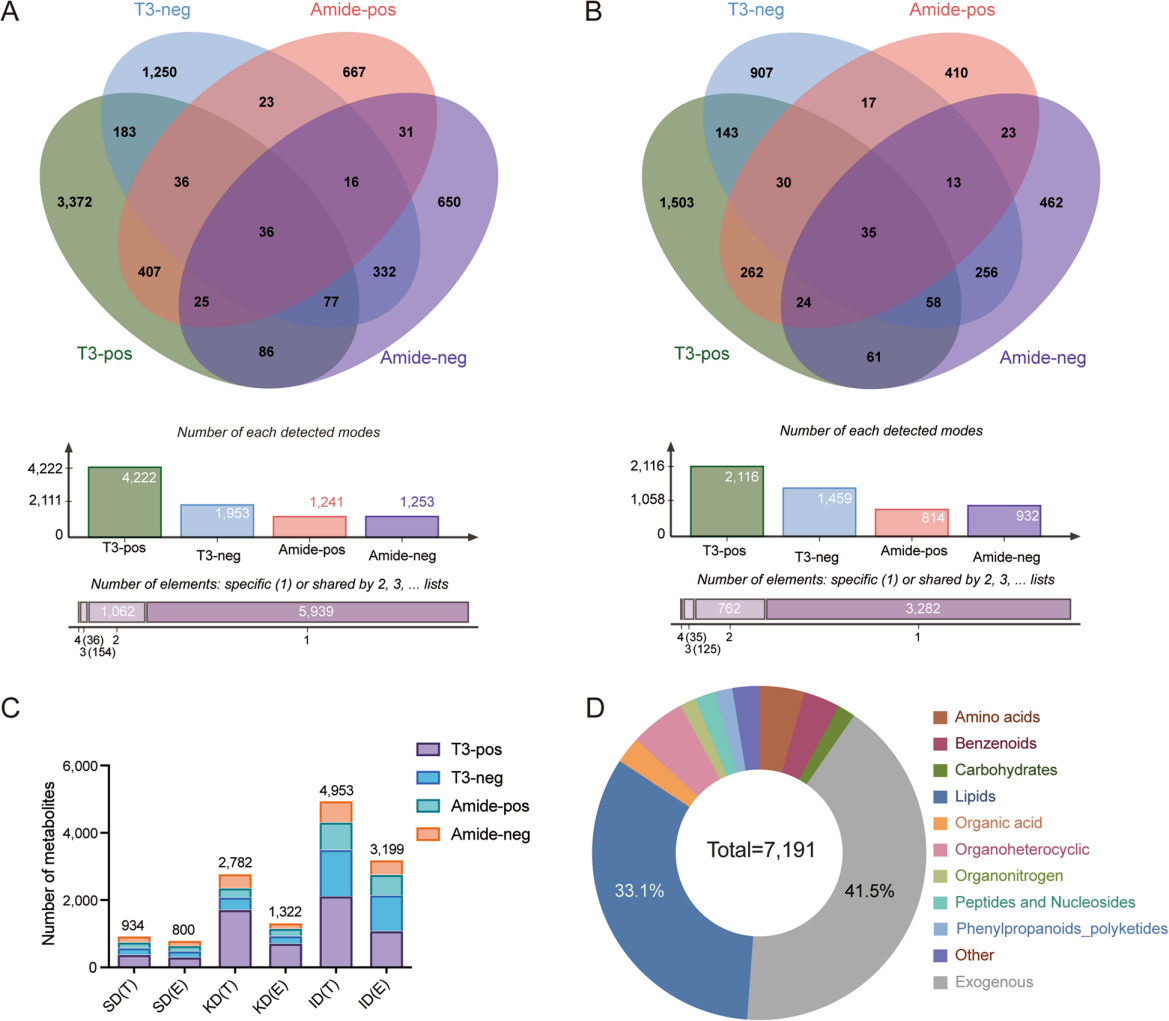
Chicken serum metabolic profiles based on QC samples. **A** Venn diagram of all metabolites identified in the four detection modes. The same metabolites were detected by multiple modes with a count of 1. **B** Venn diagram of endogenous metabolites identified in the four detection modes. The same metabolites were detected by multiple modes with a count of 1. **C** Identification of metabolites using three classes of database. SD, Standards Database; KD, KEGG Database; ID, Intergrated Database; T, Total metabolites; E, Endogenous metabolites. **D** Classification of all metabolites

In this study, non-targeted LC–MS/MS metabolomics was performed using serum sampled from 12-week-old chickens of the F16 generation of an advanced intercross line (AIL). A total of 7,191 metabolites and 4,204 endogenous metabolites were detected and identified (Fig. 1A and B, Table S1). Of these metabolites, three levels of databases were used for identification, the standard databased identified 934 metabolites of which were 800 endogenous; the KEGG database identified 2,782 metabolites of which were 1,322 endogenous; the Integrated database, identified 4,953 metabolites in total, of which 3,199 were endogenous (Fig. 1C). The identified compounds covered a wide range of biochemicals, including amino acids, benzenoids, carbohydrates, lipids, organic acids, organic heterocyclic compounds, organonitrogen, peptides and nucleosides, phenylpropanoids and polyketides, others, and exogenous compounds (Fig. 1D, Table S1).

We used the above metabolites to construct a non-targeted dataset of chicken serum metabolites using a systematic and automated approach and homemade software (OSI/SMMS) [28]. The dataset contains basic metabolite information such as molecular formula, *m/z*, retention time, primary and secondary spectral scores, and internal and external source information. For each metabolite, the first- and second-level mass spectra could be viewed, and metabolites with a combined score higher than 0.5, which is highly reliable, were retained in the dataset. Our dataset was able to help identify many more metabolites in a separate batch of muscle samples, than the traditional online database (Fig. S1).

### Metabolomic characterization of the AIL F16 population

**Fig. 2:**
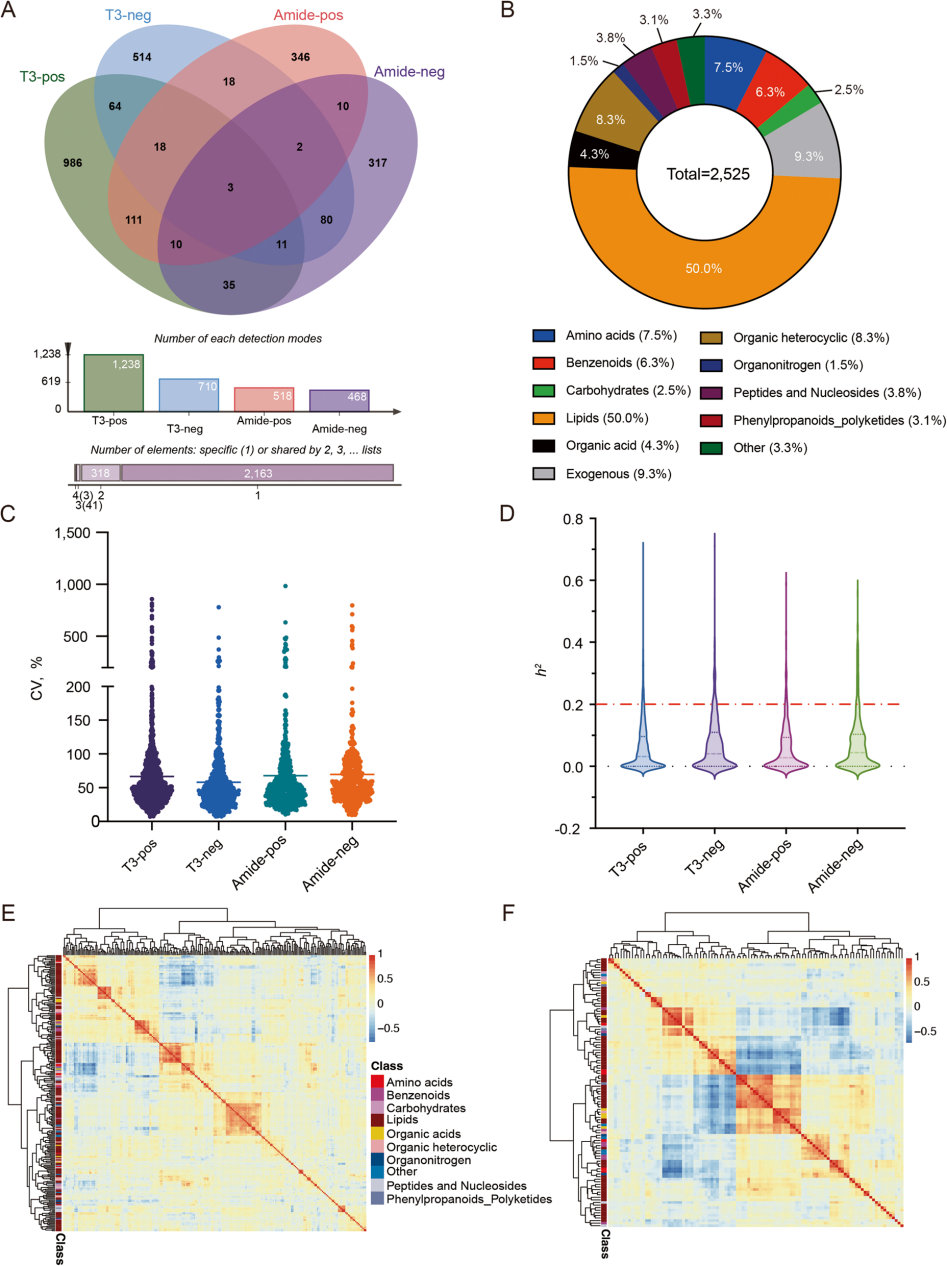
Metabolomic characterization of chicken AIL population. **A** Venn diagram of metabolites identified in the four detection modes. The same metabolites were detected by multiple modes with a count of 1. **B** Pie chart showing the percentage of each type of metabolite detected across all four modes. **C** Distribution of the values of the coefficient of variation (CV) across the four detection modes, with the mean CV indicated by a horizontal line. **D** Distribution of the broad-sense heritability (*h*^2^) of metabolites across the four detection modes, with the dashed red line representing *h*^2^ = 0.2. **E** Heat map displaying paired Pearson's correlations (*r*) between metabolites detected under the T3 modes, with metabolites classified according to a hierarchical clustering analysis based on correlations. Metabolites included in heat map had an *r* > 0.8, *P* < 0.05. **F** Heat map displaying paired Pearson’s correlations (*r*) between metabolites detected under the Amide modes, with metabolites classified according to a hierarchical clustering analysis based on correlations. Metabolites included in heat map had an *r* > 0.8, *P* < 0.05

A total of 508 chicken serum samples from the F16 generation were analyzed using a non-targeted LC–MS/MS method. After quality control, the total number of metabolic features extracted in each detection mode was 27,818, 20,274, 14,569 and 11,054 (T3-pos, T3-neg, Amide-pos and Amide-neg, respectively). Using the chicken serum metabolite dataset matching identification, 1,238, 711, 518, and 468 metabolites were identified in four modes for a total of 2,525 metabolites (Fig. 2A, Table S2). The metabolites were consisted of amino acids (7.52%), benzenoids (6.34%), carbohydrates (2.53%), exogenous compounds (9.31%), lipids (49.94%), organic acids (4.32%), organic heterocycles (8.32%), organonitrogen (1.47%), peptides and nucleosides (3.84%), phenylpropanoids and polyketides (3.09%), and other metabolites (3.33%) (Fig. 2B, Table S2). These metabolites are involved in multiple important metabolic pathways supporting key cellular processes.

Principal component analysis (PCA) of all features showed no population stratification of metabolites detected in the same mode (Fig. S2). The levels of metabolite accumulation in the samples varied considerably, which allowed for efficient analysis of their genetic structure. In the AIL population, the mean genetic coefficients of variation (CV) of these metabolites were 66.7%, 58.0%, 67.8% and 69.5% across the four modes (Fig. 2C, Table S3). The substances with the maximum mean CV among the different modes, with amino acids (81.9% in T3-pos), benzenes (72.6% in T3-neg), organonitrogen (131.7% in Amide-pos mode) and 95% in peptides and nucleotides in Amide-neg mode. 5,6-Dihydroxyindole had the highest CV value (857.9%) of all metabolites.

The broad sense heritability (*h*^2^) distribution of metabolic traits showed that more than 7.8% of the metabolites showed heritability above 0.2 (Fig. 2D, Table S4). In Amide-neg mode, the compound with the highest heritability was 2'-O-Methyluridine, which had an *h*^2^ of 0.56. Under the other three modes (T3-pos, T3-neg and Amide-pos), ceramide (Cer 36:3) had the highest heritability, with an *h*^2^ of 0.69, 0.71 and 0.59, respectively. These results show that genetic factors affect the heritability of the metabolites.

Metabolite correlation analysis was performed using Pearson's correlation coefficient, and heat maps were plotted by screening metabolites with high metabolite correlation coefficients (*r* > 0.8, *P* < 0.05; Fig. 2E and F). The results showed that lipids can be divided into several distinct clusters, while different metabolite species were also present in the clusters. This indicates that the overall similarity of metabolites of the same species is higher, and that metabolites in the same metabolic pathway are more likely to cluster.

### Metabolome genome-wide association study (mGWAS)

**Fig. 3:**
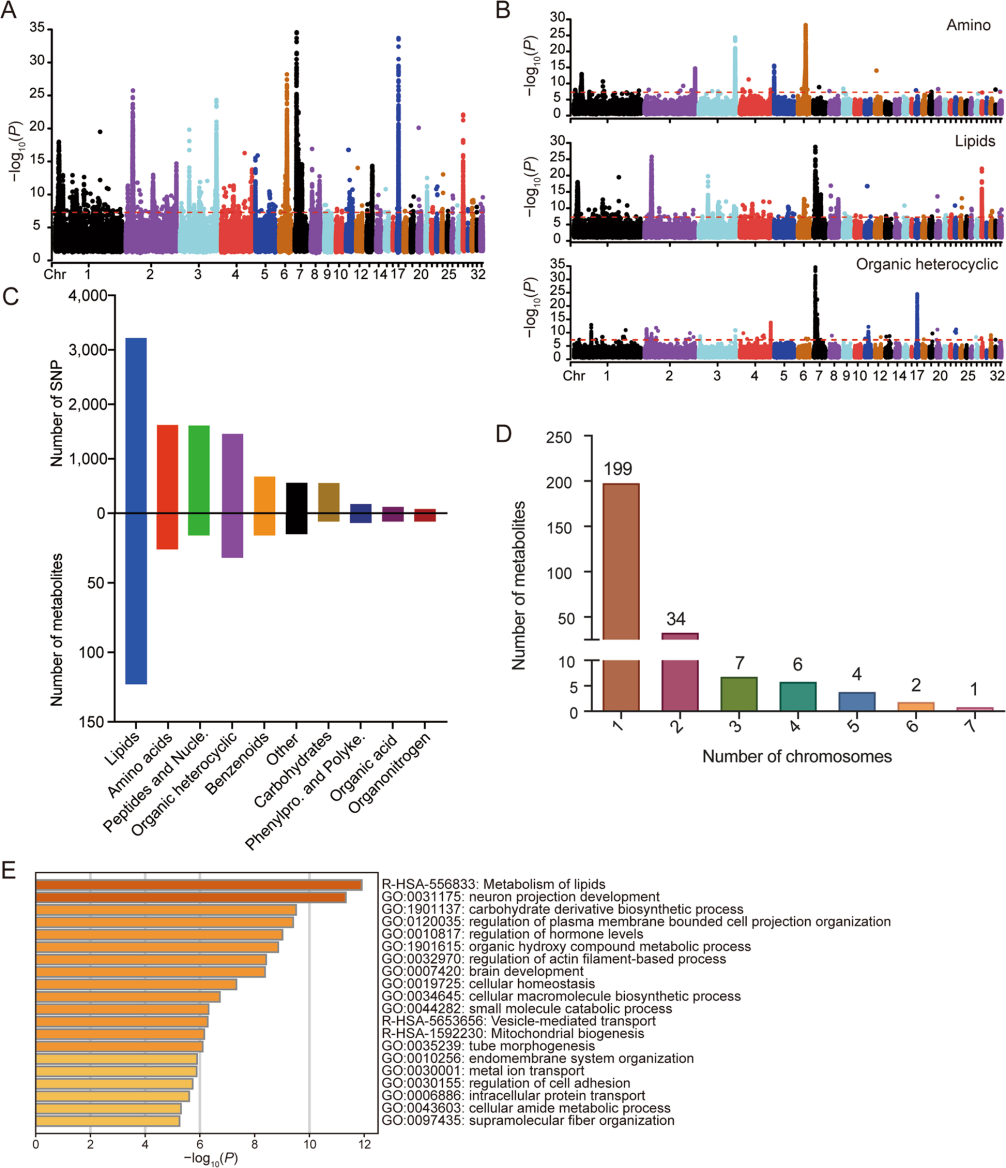
Overview of metabolites and SNPs identified in the mGWAS. **A** Chromosomal distribution of all SNPs, where the significance threshold is −log_10_(*P*) > 7.29. **B** Chromosomal distribution of SNPs in different metabolites classes, where the significance threshold is −log_10_(*P*) > 7.29. **C** The number of SNPs mapped by different classes of metabolites. Petides and Nucle., Petides and Nucleosides; Phenylpro. and Polyke., Phenylpropanoids and polyketides. **D** Statistics on the number of chromosomes distributed by the same metabolite SNPs. **E** Functional enrichment of all SNP-annotated genes

The AIL population was sequenced using a low-coverage whole-genome sequencing strategy (LCS), and approximately 960 K SNPs were obtained after quality control for genomic coverage ranging from 0.11 to 2.60 × , the mean coverage was 0.91 ± 0.23 × (mean ± SD). Of the 2,935 metabolites (362 metabolites present in at least two detection modes), 253 metabolites had mGWAS signals, with 10,061 SNPs reaching the significance threshold [−log_10_ (*P*) > 7.29] (Fig. 3A, Table S5). These SNP loci were widely distributed throughout the genome, mainly concentrated on chr1, chr2, chr3, chr7, and chr17 (8,152/10,061 = 81.0%) (Fig. 3A, Fig. S3, Table S5). The mGWAS signal was identified at different locations in the genomes of different compound species (Fig. 3B, Fig. S4). The number of lipid-related SNPs (3,217) was the highest among all categorized metabolites, followed by amino acids, peptides, and nucleosides (Fig. 3C). For each metabolite reaching the threshold SNPs chromosome location, of which, 78.7% (199/253) metabolites had signal on only one chromosome, 21.3% (54/253) metabolites had signal on multiple chromosomes, and *L*-tyrosine methyl ester had signal on 7 chromosomes (Fig. 3D). All SNPs that reached the significant threshold corresponded annotated to 1,689 genes, mainly pathways including lipid metabolism, neuronal development, and regulation of hormone levels (Fig. 3E).

### Identification of candidate genes

**Fig. 4:**
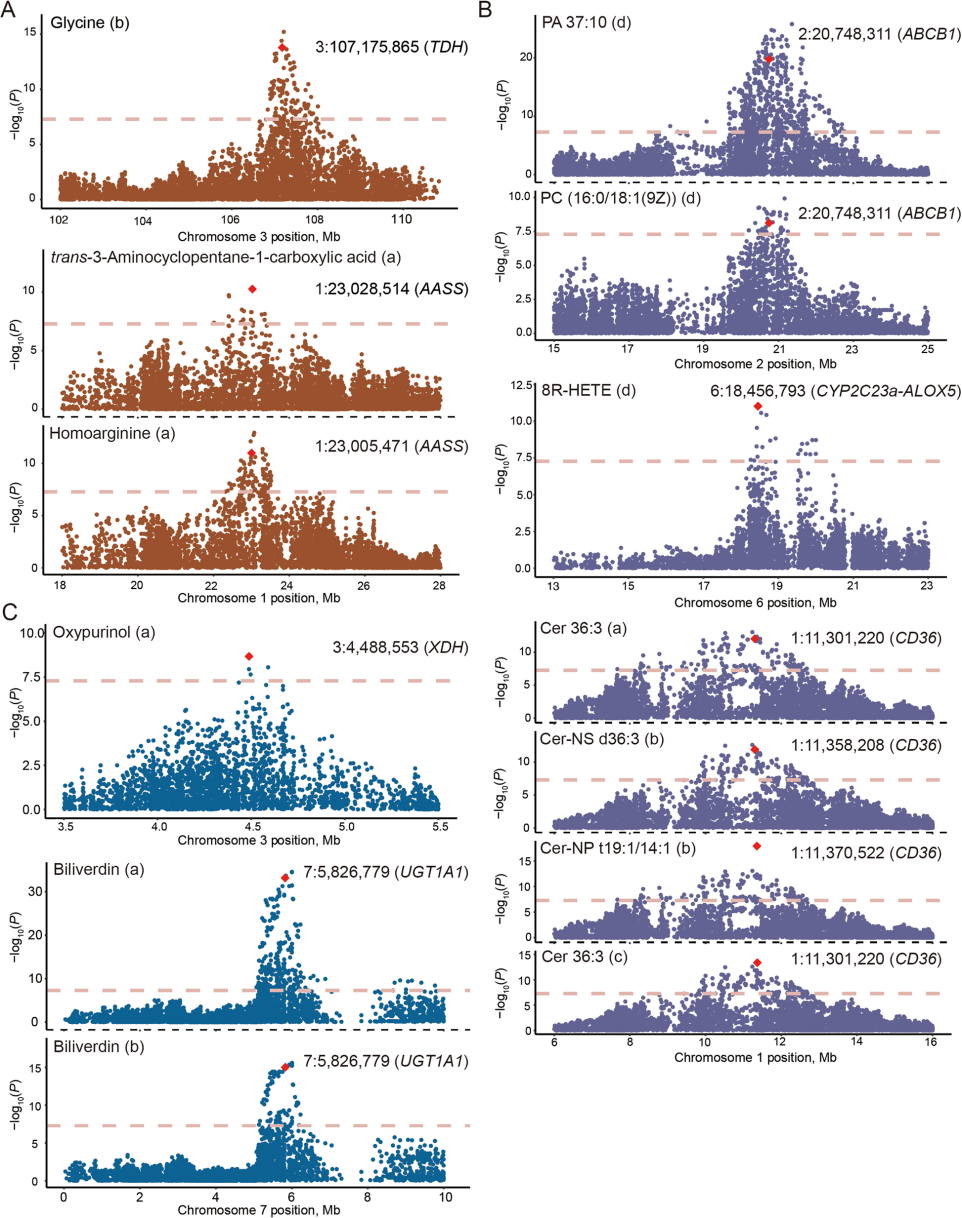
Mapping of SNP-annotated genes associated with metabolites. **A** Annotated gene display of amino acid analogues corresponding to SNPs. **B** Annotated gene display of lipid analogues corresponding to SNPs. **C** Annotated gene display of organic heterocyclic analogues corresponding to SNPs. Key SNPs are marked with a red diamond, and the letters a, b, c and d following metabolite names are the detection modes, T3-pos, T3-neg, Amide-pos, Amide-neg, respectively

Based on metabolite and gene function annotation information, we identified a number of genes associated with multiple pathways of metabolite synthesis, metabolism, and regulation (Table 1). In this study, 26 amino acids and their derivatives had GWAS signals; 1,622 SNPs were significantly associated with these compounds, mainly on chr3 and chr6 (Fig. 3B, Table S5). The main metabolic pathways involved included glycine, serine, and threonine metabolism, glyoxylate and dicarboxylate metabolism, and arginine and proline metabolism (Fig. S5). Eight genes containing SNPs significantly associated with metabolites were related to amino acid synthesis and metabolism. For example, the SNP (3:107,175,865) was significantly associated with glycine (*P* = 1.64E−14, Fig. 4A), and the annotated gene for this SNP locus was *TDH* (encoding *L*-threonine dehydrogenase); *trans*-3-aminocyclopentane-1-carboxylic acid (1:23,028,514, *P* = 5.38E−11) and homoarginine (1:23,005,471, *P* = 1.04E−11) showed a significant association with two SNPs located in a gene on chromosome 1, encoding a bifunctional enzyme that catalyzes the first two steps of the mammalian lysine degradation pathway (aminoadipate-semialdehyde synthase, *AASS*; Fig. 4A).

**Table 1:** Summary of genes associated with metabolite synthesis, metabolism and regulation in the mGWAS

Gene name	Chr	Position	Metabolite name	Ref/Alt	*P*-value	Gene description	Metabolite ID^1^
Amino acids							
*AASS*	1	23,028,514	*trans*-3-Aminocyclopentane-1-carboxylic acid	G/A	5.38E−11	Aminoadipate-semialdehyde synthase	a2192
	1	23,005,471	Homoarginine	A/T	1.04E−11		a6139
*PYCR3*	2	148,895,024	*D*-2-Aminoadipic acid	C/T	2.68E−13	Pyrroline-5-Carboxylate Reductase 3	a2916
*FDFT1*	3	107,355,672	*L*-2-Amino-6-oxoheptanedioate	A/G	6.92E−15	Farnesyl-Diphosphate Farnesyltransferase 1	a6166
*MSRA*	3	106,645,100	*L*-2-Amino-6-oxoheptanedioate	A/G	2.76E−10	Methionine Sulfoxide Reductase A	a6166
	3	107,535,427	N-oleoyl threonine	C/T	7.75E−12		a18373
*TDH*	3	107,175,865	Glycine	T/C	1.64E−14	*L*-Threonine Dehydrogenase (Pseudogene)	b23
*MTMR4*	4	14,104,422	*L*-Serine	C/T	3.78E−08	Myotubularin Related Protein 4	c356
*GOT1*	6	23,099,727	N6-Methyl-*L*-lysine	T/C	2.52E−19	Glutamic-Oxaloacetic Transaminase 1	a4060
*HOGA1*	6	23,275,981	N6-Methyl-*L*-lysine	A/G	3.83E−20	4-Hydroxy-2-Oxoglutarate Aldolase 1	a4060
Lipids							
*CD36*	1	11,301,220	Cer 36:3	T/C	9.05E−13	Cluster of differentiation 36	a26248
	1	11,358,208	Cer-NS d36:3	G/A	1.33E−18		b21105
	1	11,370,522	Cer-NP t19:1/14:1	A/C	4.19E−14		b21776
	1	11,301,220	Cer 36:3	T/C	1.31E−12		c11086
*RORA*	1	121,625,308	17α,21-Dihydroxypregnenolone	A/G	1.93E−08	RAR Related Orphan Receptor A	a16236
*ABCB1*	2	20,748,311	PA 37:10	G/A	1.87E−20	ATP Binding Cassette Subfamily B Member 1	d14475
	2	20,748,311	1-Hexadecanoyl-2-(9Z-octadecenoyl)-sn-glycero-3-phosphoserine (PC(16:0/18:1(9Z)))	G/A	7.58E−09		d14771
*CUBN*	2	19,974,415	(3S,3'R,5R,6R)-7',8'-Didehydro-3,6-epoxy-5,6-dihydro-beta, beta-carotene-3',5-diol	C/T	4.11E−10	Cubilin	d13798
	2	19,974,415	PA 37:10	C/T	3.19E−15		d14475
*HACD2*	2	19,684,787	PA 37:10	T/A	4.30E−12	3-Hydroxyacyl-CoA Dehydratase 2	d14475
*OLAH*	2	20,632,976	PA 37:10	A/G	8.80E−24	Oleoyl-ACP Hydrolase	d14475
	2	20,812,193	PS 36:2	C/A	8.98E−09		d14886
*MTMR9*	3	107,155,447	PC 35:3	C/T	2.24E−09	Myotubularin Related Protein 9	b24244
*SNX17*	3	104,426,364	SM 34:0	G/T	7.63E−09	Sorting Nexin 17	c14637
*HPGDS*	4	37,173,242	Corticosterone	A/C	4.43E−12	Hematopoietic Prostaglandin D Synthase	d9959
*GALC*	5	43,096,098	Phosphatidylethanolamine 18:2–18:2	T/C	3.66E−10	Galactosylceramidase	b23510
*NPC2*	5	38,143,267	Plasmenyl-PC 34:2	G/A	3.75E−08	NPC Intracellular Cholesterol Transporter 2	a32135
*CYP2C23a-ALOX5*	6	18,456,793	8(R)-Hydroxy-(5Z,9E,11Z,14Z)-eicosatetraenoic acid (8R-HETE)	G/A	9.44E−12	Cyclooxygenases, and cytochrome P450s-lipoxygenases	d9222
*AGAP1*	7	5,491,791	18-acetoxy-1α;,25-dihydroxyvitamin D3	G/A	4.36E−08	ArfGAP With GTPase Domain, Ankyrin Repeat And PH Domain 1	a22758
	7	5,223,906	4α-Methylzymosterol-4-carboxylate	G/A	6.50E−13		d12116
*AHR2*	7	6,569,111	4α-Methylzymosterol-4-carboxylate	A/G	2.96E−11	Aryl Hydrocarbon Receptor	d12116
*PLPP3*	8	26,062,752	PE (2:0/19:0)	G/A	5.11E−09	Phospholipid Phosphatase 3	d13432
*PRKAA2*	8	26,124,947	13E,15E,18Z,20Z-pentacosatetraen-11-ynyl acetate	A/G	1.21E−08	Protein Kinase AMP-Activated Catalytic Subunit Alpha 2	a19217
	8	26,131,440	PE (2:0/19:0)	G/A	1.17E−08		d13432
*GPAT2*	22	5,343,878	LysoPC 20:4	C/T	2.91E−08	Glycerol-3-Phosphate Acyltransferase 2, Mitochondrial	a26448
*PIAS4*	28	1,553,948	Monoanhydroescholtzxanthin	C/T	5.06E−11	Protein Inhibitor of Activated STAT 4	c10859
Organic heterocyclic							
*XDH*	3	4,488,553	Oxypurinol	T/C	8.79E−09	Xanthine Dehydrogenase	a3598
*UGT1A1*	7	5,826,779	Biliverdin	C/T	3.70E−19	UDP glucuronosyltransferase 1 family, polypeptide A1	a27158
	7	5,826,779	Biliverdin	C/T	6.92E−34		b21472

^1^The letters a, b, c and d preceding under metabolite ID numbers represent the 4 detection modes: T3-pos, T3-neg, Amide-pos and Amide-neg, respectively

A total of 123 lipids had mGWAS signals, mainly glycerophospholipids, steroids, sphingolipids, and other substances (Fig. S6). A total of 3,217 SNPs of lipids reached the significance threshold, with signals mainly from chr1, chr2 and ch7 (Fig. 3B). The SNPs were annotated to 19 genes related to lipid metabolism (Table 1), mainly involving fatty acid metabolism, medium-chain fatty acid metabolic processes, and linoleic acid metabolic processes. The SNP (2:20,748,311) located within the ATP- binding cassette subfamily B member 1 gene (*ABCB1*) was associated with 1-hexadecanoyl-2-(9Z-octadecenoyl)-sn-glyce ro-3-phosphoserine (PC(16:0/18:1(9Z))) (*P* = 7.58E−09) and PA 37:10 (*P* = 1.87E−20, Fig. 4B); 8(R)-hydroxy-(5Z,9E,11Z,14Z)-eicosatetraenoic acid (8R-HETE) is a metabolite of arachidonic acid, which reached a significance threshold SNP (6:18,456,793) located in the intergenic region of *ALOX5* and *CYP2C23a* genes (*P* = 9.44E−12, Fig. 4B). Arachidonic acid can be oxygenated by a variety of enzymes, including lipoxygenases (*ALOX5*, etc.), cyclooxygenases, and cytochrome P450s (*CYP2C23a*), and can be converted to a complex mixture of oxygenated products as a result of lipid peroxidation. The three SNPs (1:11,301,220, *P* = 9.05E−13; 1:11,358,208, *P* = 1.33E−18; 1:11,370,522, *P* = 4.19E−14; 1:11,301,220, *P* = 1.31E−12), which were significantly associated with the sphingolipid metabolite Cer were located within the *CD36* molecule gene (*CD36*), the most important transmembrane glycoprotein mediating the uptake of oxidized LDLs (Fig. 4B).

In addition to these amino acids and lipids, oxypurinol an inhibitor of xanthine oxidase, a metabolite of allopurinol. *XDH* (Xanthine dehydrogenase) is a key enzyme involved in purine degradation and is related to hydroxypurinol. The SNP located in *XDH* (3:4,488,553) was significantly associated with oxypurinol (*P* = 8.79E−09, Fig. 4C). The SNP (7:5,826,779) in bilirubin was detected in both T3-pos (*P* = 3.70E−19) and T3-neg (*P* = 6.92E−34), reached a significance threshold, and was located within UDP glucuronosyltransferase family 1 member A1 gene (*UGT1A1*), which is associated with cholesterol synthesis (Fig. 4C).

## Discussion

### Chicken metabolite dataset and metabolite identification

This study established the first large-scale serum metabolomic profile in chickens. A total of 7,191 metabolites were identified using non-targeted metabolomics; these were integrated to form a chicken serum metabolite public reference dataset that can provide a reference for future chicken metabolomics studies.

The identification of unknown metabolites is an urgent problem in metabolomics research. In this study, more than 70,000 MS features were obtained by peak extraction and filter correction; however no more than 5% of the metabolites could be identified because of the limitations of the current metabolite database, which greatly limited the subsequent tests. Metabolite structure identification based on metabolic reaction networks can largely identify unknown metabolites [36]; alternatively, a widely targeted metabolomics strategy combining the high resolution and wide coverage of non-targeted technologies with the accuracy benefits of targeted MRM technologies could improve the efficiency of metabolomic assays [37]. Once the method is established, all samples can be assayed using a triple quadrupole liquid mass spectrometry instrument to obtain more accurate quantitative information [38]. A variety of widely targeted metabolomics strategies have been published, such as the full scan and ddMS2 mode combination method [39], SWATH-MS method [40], and multiple ion monitoring enhanced product ion method (MIM-EPI) [41]. We obtained all the information on non-targeted metabolomics of chicken serum, and we will continue to select and validate MRM ion pairs to establish a widely targeted metabolomics method for chicken serum in the future.

The reproducibility of high-throughput metabolomics metabolite detection was the basis for the subsequent experiments [42]. In the present study, only peaks that were present in more than three samples and responded to signals exceeding 15,000 (prefilter = c (3, 15,000)) were retained in the peak extraction stage. Also, during subsequent data processing, the mass spectrometry features present in more than 50% of the QC samples were retained, and the missing mass spectrometry features in more than 80% of the samples were removed. Finally, only the mass spectral features with CV values < 50% in the QC samples were analyzed to avoid interference caused by false-positive peaks to the maximum extent possible. With the subsequent metabolomic detection experiments on the remaining serum samples of the AIL population, metabolites that were not reproducible in both batches will be further screened to obtain more reliable analytical conclusions.

### Heritability of animal metabolites

Metabolites with moderate levels of heritability have the potential to serve as biomarkers for genetic selection. Plant secondary metabolites have higher heritability than primary metabolites, and flavonoid metabolites can have heritability greater than 0.7 [6]. The literature suggests that in humans, approximately 50% of phenotypic variation in metabolite levels is genetically induced, but heritability estimates vary across metabolite classes [43]. Compared to plants, which are protected against external stimuli by secondary metabolites [44], animals are exposed to more diverse environmental stimuli, such as living environment, diet, and drugs [45, 46]. In our study, the heritability of peptides and nucleosides was higher than that of other metabolite classes in all four detection modes, this suggests their potential as biomarkers for genetic selection. A total of 34.2% of the metabolite heritability was zero, mainly influenced by environmental factors; in contrast, a study of plasma metabolites in beef cattle found that 22 of 33 metabolites had zero or negligible heritability, the non-heritable status of these metabolites may be used as a guide to animal management [17]. In our study, 7.8% of metabolite heritability was greater than 0.2. Cer 36:3 reached high heritability levels (> 0.59 in all three assay modes), and the heritability of hypoxanthine (0.38) was moderate. Studies have shown that the heritability values of long-chain polyunsaturated fatty acids in pork are usually above 0.50 [47] and that hypoxanthine is one of the highest heritability metabolites among plasma metabolites in young healthy pigs [46].

### mGWAS and candidate genes

This study reported mGWAS signals for 253 metabolites representing the largest chicken serum metabolite association study to date. Metabolites and their associated loci broadly cover representatives of all major metabolic pathways, providing a comprehensive picture of how genetic variation affects blood metabolic homeostasis in chickens. We identified two candidate genes, *TDH* and *AASS* associated with amino acid metabolites, and another two candidate genes, *ABCB1*, *CD36* associated with lipids (Table 2) [48–54]. Amino acids are essential for animal growth and development. In the chicken feeding process, different types and proportions of amino acids are usually added to the diet to suit the growth needs of chickens at different stages [55]. Lipids are important biomolecules in animals and have a variety of biological functions, including energy storage, signal recognition, and immunity [56]. Lipid metabolism is closely related to the maintenance of the dynamic energy balance and physiological functions in broilers [18].

**Table 2:** Summary of key gene and metabolite functions in the mGWAS

Gene name	SNP (Ref/Alt)	Metabolite name^1^	Metabolite content in different genotypes (log10, mean ± SD)	Gene and metabolite function description
*TDH*	3:107,175,865 (T/C)	Glycine	TT: 15.081 ± 0.477	Mitochondrial threonine dehydrogenase (TDH) enzyme to catabolize threonine into glycine and acetyl-CoA in mouse ES cells [48];
			TC/CT: 14.733 ± 0.419	TDH catalyzes the conversion of 2-amino-3-oxobutyrate to acetyl CoA and glycine [49];
			CC: 14.600 ± 0.416	
*AASS*	1:23,005,471 (A/T)	Homoarginine	AA: 18.664 ± 0.483	Synthesis of *L*-homoarginine (hArg) from Arg and *L*-lysine in animals and humans [50];
			AT/TA: 18.216 ± 0.406	*AASS* catalyzes the first two steps in the mammalian lysine degradation pathway;
			TT: 18.042 ± 0.807	
*ABCB1*	2:20,748,311 (G/A)	PA 37:10	GG: 12.528 ± 0.731	ABC transporter protein is a lipid transporter protein [51];
			GA/AG: 13.045 ± 0.723	ABCB1 transports phospholipids [52];
			AA: 13.834 ± 0.878	
		1-Hexadecanoyl-2-(9Z-octadecenoyl)-sn-glycero-3-phosphoserine (PC(16:0/18:1(9Z)))	GG: 16.868 ± 0.510	
			GA/AG: 17.074 ± 0.496	
			AA: 17.444 ± 0.469	
*CD36*	1:11,301,220 (T/C)	Cer 36:3 (a)	TT: 14.447 ± 0.709	Cer 36:3 is a ceramide, members of the class of compounds known as sphingolipids;
			TC/CT: 15.143 ± 0.522	CD36 is a number of key proteins involved in fatty acid uptake [53];
			CC: 15.225 ± 0.585	
		Cer 36:3 (c)	TT: 15.723 ± 0.452	
			TC/CT: 16.160 ± 0.380	
			CC: 16.199 ± 0.475	
	1:11,358,208 (G/A)	Cer-NS d36:3	GG: 13.856 ± 0.647	
			GA/AG: 14.579 ± 0.473	
			AA: 14.734 ± 0.492	
	1:11,370,522 (A/C)	Cer-NP t19:1/14:1	AA: 11.926 ± 1.069	
			AC/CA: 12.801 ± 0.815	
			CC: 12.979 ± 0.774	
*UGT1A1*	7:5,826,779 (C/T)	Biliverdin (a)	CC: 16.607 ± 0.695	UGT1A1 encodes an enzyme with bilirubin glucuronidating activity;
			CT/TC: 15.991 ± 0.601	Bilirubin is derived from biliverdin [54]
			TT: 15.898 ± 0.543	
		Biliverdin (b)	CC: 14.646 ± 0.410	
			CT/TC: 13.961 ± 0.453	
			TT: 13.746 ± 0.519	

^1^The letters a, b and c following the metabolite names represent the 3 detection modes: T3-pos, T3-neg and Amide-pos, respectively

Traditional GWAS have identified a large number of SNPs and candidate genes related to economic traits in chickens; for example, the end of chromosome 1 contains a QTL with a sizable effect on growth traits such as chicken body weight [13, 24], and a large number of SNPs related to chicken meat quality traits [57], egg-laying traits [58] and fat deposition have been identified [59]. However, identifying which of these many SNPs represent the main causative mutations has proven difficult. Higher-resolution association analysis using simple metabolic phenotypes instead of complex comprehensive phenotypes can provide more refined localization. For instance, the amount of fat deposited in livestock and poultry is an important economic factor because it is associated with meat quality and feed conversion rate (FCR). Here, we identified 123 lipids with mGWAS signals, of which the annotated genes *PLK3* and *SLC16A1* (located in this QTL region) were associated with abdominal adipogenesis in chickens [11, 60], and *ITGA8* is a core gene associated with epigenetic energy in chickens [61] (Table S5). A previous study reported that multiple causative mutations cumulatively contribute to this major QTL, which may be explained by the genes playing their respective roles in different metabolic pathways affecting chicken growth traits.

This AIL population is a very valuable experimental resource, and our GWAS analysis in F9 not only reproduced the results of F2, but also further increased the localization accuracy [13]. This is the first mGWAS in an AIL line, and the related metabolic phenotypes were also obtained for the first time. The significant SNPs will be of great breeding value, and we believe that the repeated experiments in subsequent generations can be used to further reproduce and confirm the current results and further improve the precision.

## Conclusions

We performed a large-scale serum non-targeted metabolomic assay on a chicken AIL population to provide the first comprehensive characterization of the chicken serum metabolic profile. Containing 7,191 metabolites, the first non-targeted in-house metabolite database of chickens was established. The mGWAS analysis was performed with the SNP dataset obtained from low-coverage sequencing and metabotypes, and a total of 10,061 SNPs for 253 metabolites were reported as genome-wide significant [−log_10_ (*P*) > 7.29]. GWAS loci were mainly concentrated in chr1, chr2, chr3, chr7 and chr17. A large number of candidate genes related to the synthesis, metabolism, and regulation of this class of metabolites were identified in amino acids, lipids, and organic heterocycles. This study provides a comprehensive picture of how genetic variation affects blood metabolic homeostasis in chickens and provides a foundation for future studies on the use of metabolic phenotypes to understand complex economic traits in animals.

## Supplementary Information

**Additional file 1: Fig. S1.** In-house non-targeted database of chicken serum metabolites. **Fig. S2.** Principal component analysisof all features on four detected modes. **Fig. S3.** Number of GWAS signaling metabolites on each chromosome. **Fig. S4.** Manhattan plot showing GWAS signals of another 7 classes of metabolites. **Fig. S5.** Analysis of the amino acid metabolites pathwayin the metabolites of GWAS signal using MetaboAnalyst. **Fig. S6.** Classification of lipids in the metabolites of GWAS signal using MetaboAnalyst.

**Additional file 2: Table S1.** Segmented QC samples identified to metabolites under four detection modes. **Table S2.** Non-targeted metabolites of chicken serum. **Table S3.** The statistical results of coefficient of variation. **Table S4.** The statistical results of broad-sense heritability. **Table S5.** All significant SNP loci of GWAS signaling metabolites > 7.29.

**Additional file 3: Table S6.** Metabolic phenotype QTL intervals.

## Data Availability

The datasets produced and/or analyzed during the current study are available from the corresponding author on reasonable request.

## Abbreviations

AIL: Advanced intercross line
CV: Coefficient of variation
GWAS: Genome-wide association studies
HB: Huiyang bearded chicken
HQLA: High-quality chicken Line A03
LCS: Low-coverage sequencing
mGWAS: Metabolome genome-wide association study
MIM-EPI: Multiple ion monitoring enhanced product ion
OSI/SMMS: Systematic and automated approach and homemade software
PCA: Principal component analysis
QC: Quality control
