# Supplementary material for: Serum metabolic profile and metabolome genome-wide association study in chicken
Source: J Anim Sci Biotechnol. 2023 May 4;14:69. doi: 10.1186/s40104-023-00868-7 (PMC10158329; doi:10.1186/s40104-023-00868-7)
Supplement: Supplementary file 1 — Additional file 1: Fig. S1. In-house non-targeted database of chicken serum metabolites. Fig. S2. Principal component analysisof all features on four detected modes. Fig. S3. Number of GWAS signaling metabolites on each chromosome. Fig. S4. Manhattan plot showing GWAS signals of another 7 classes of metabolites. Fig. S5. Analysis of the amino acid metabolites pathwayin the metabolites of GWAS signal using MetaboAnalyst. Fig. S6. Classification of lipids in the metabolites of GWAS signal using MetaboAnalyst. [file 40104_2023_868_MOESM1_ESM.docx]

**Supplementary material**


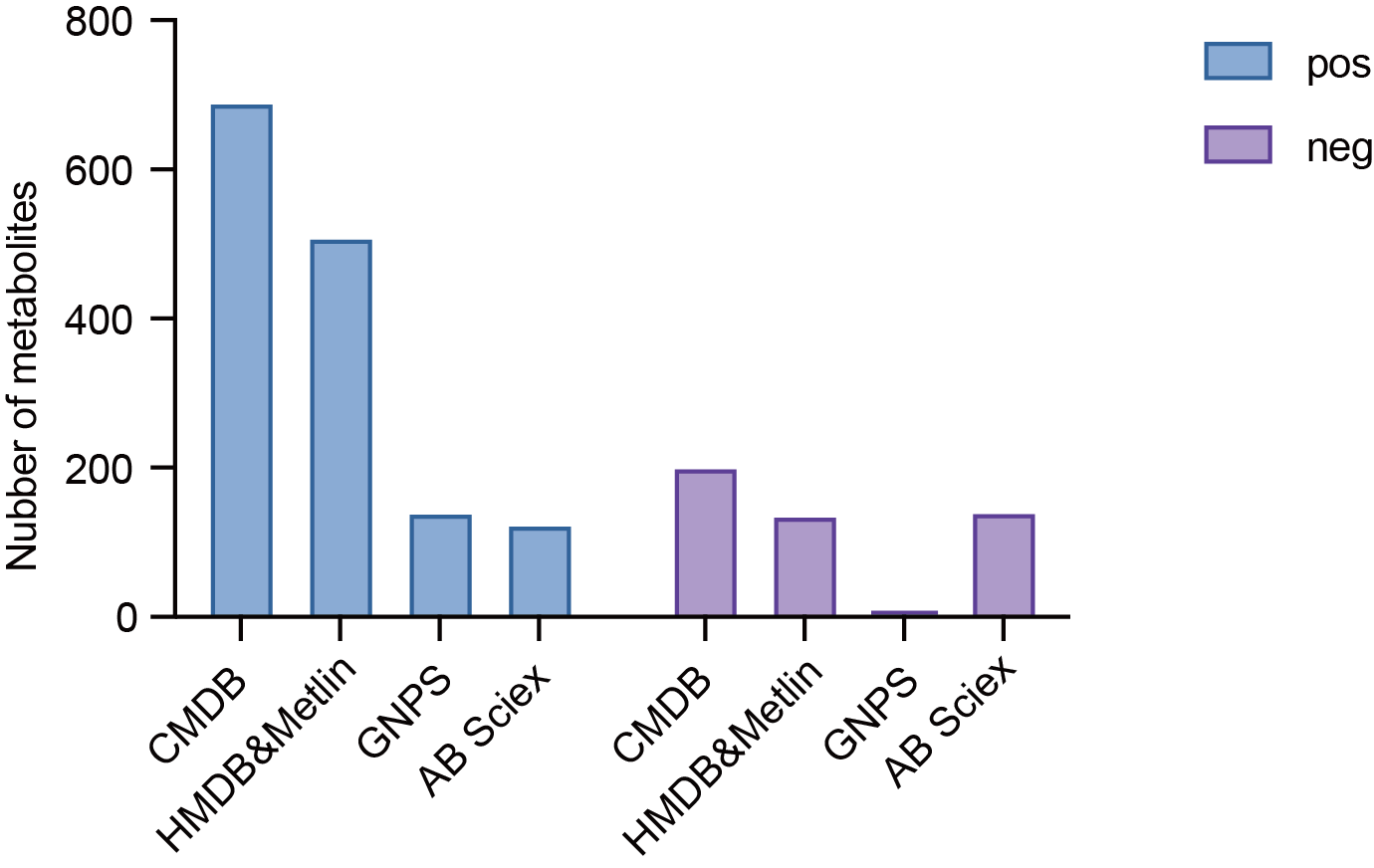


**Fig. S1** In-house non-targeted database of chicken serum metabolites. Metabolite identification using different databases for non-targeted LC-MS/MS data from chicken meat. CMDB, chicken metabolites database (our in-house chicken serum metabolites database); GNPS, Global natural products social molecular networking (a web-based mass spectrometry ecosystem); AB Sciex, Metabolite identification database embedded in AB sciex software


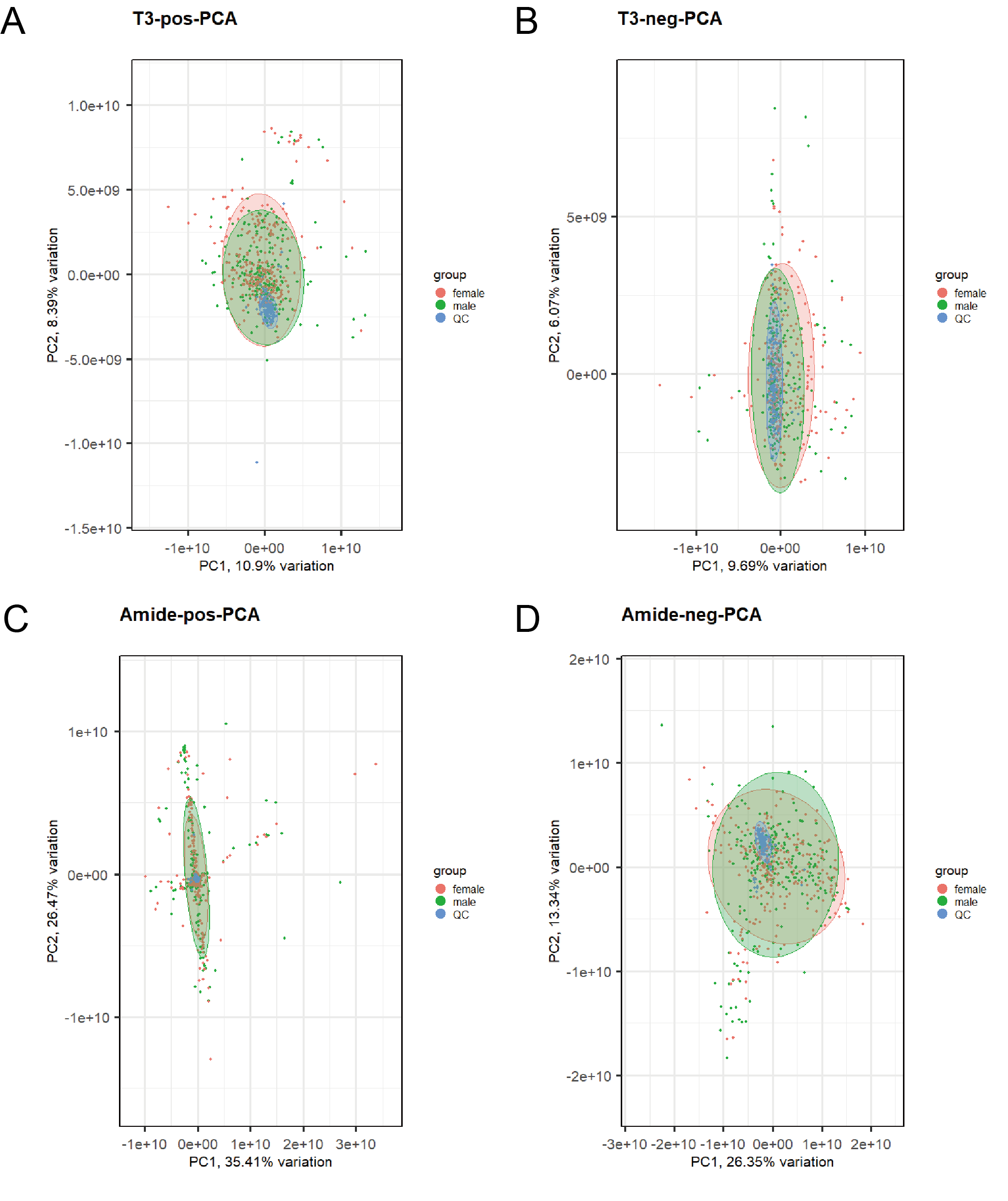


**Fig. S2** Principal component analysis (PCA) of all features on four detected modes

**Fig. S3** Number of GWAS signaling metabolites on each chromosome

**A**

**Benzennoids**

**
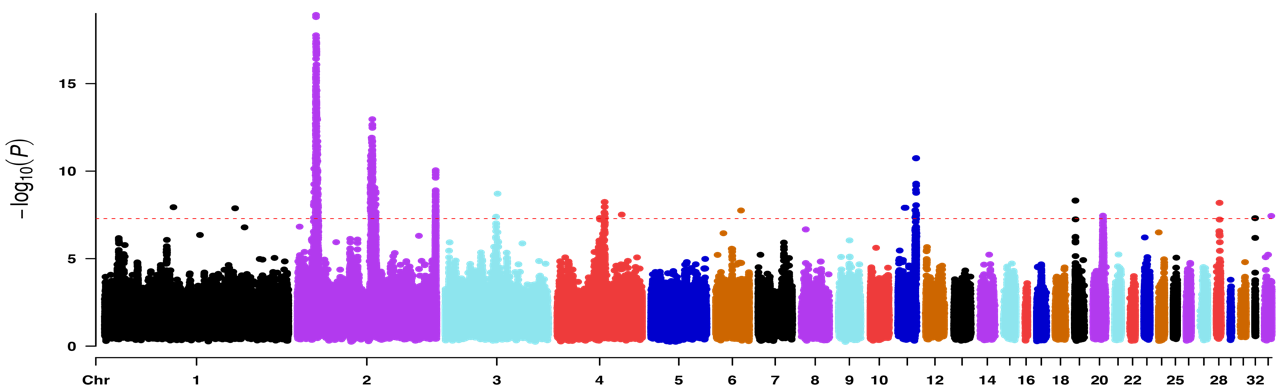
**

**B**

**Carbohydrates**

**
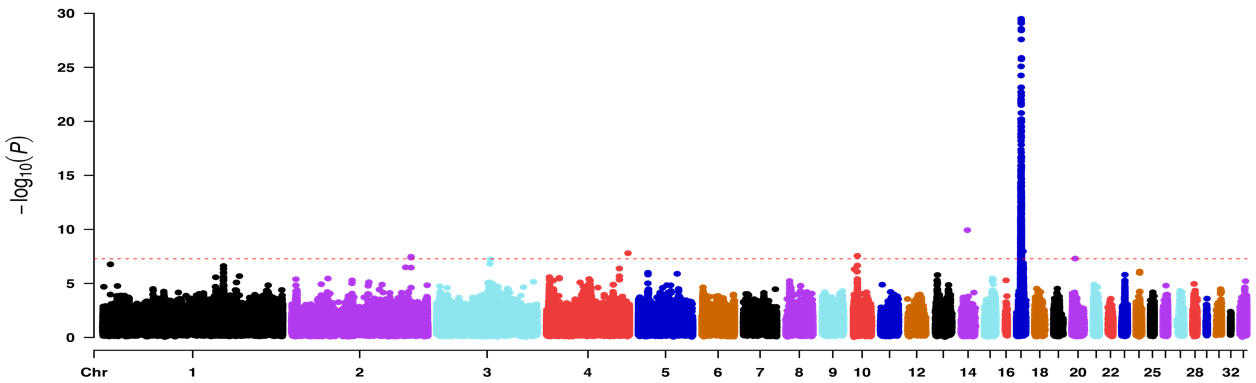
**

**C**

**Organic acids**

**
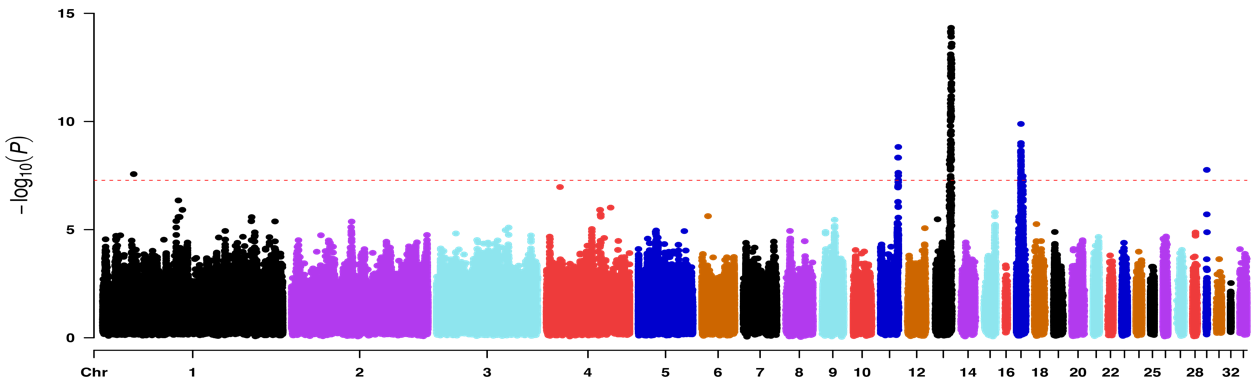
**

**D**

**Organonitrogen**

**
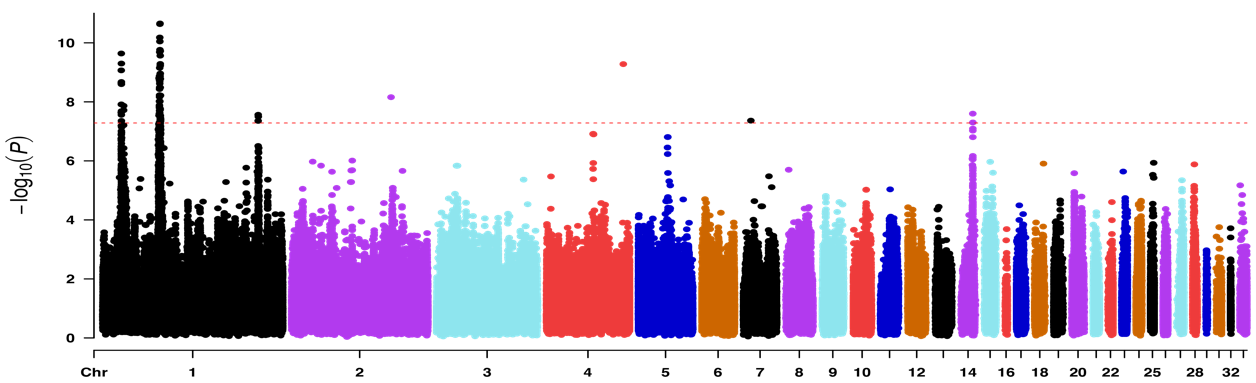
**

**E**

**Peptides and Nucleosides**

**
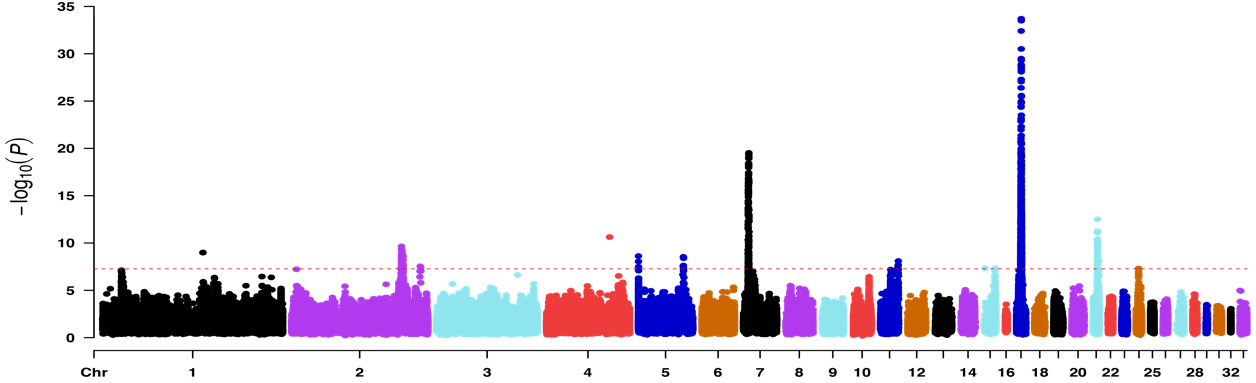
**

**F**

**Phenylpropanoids and polyketides**

**
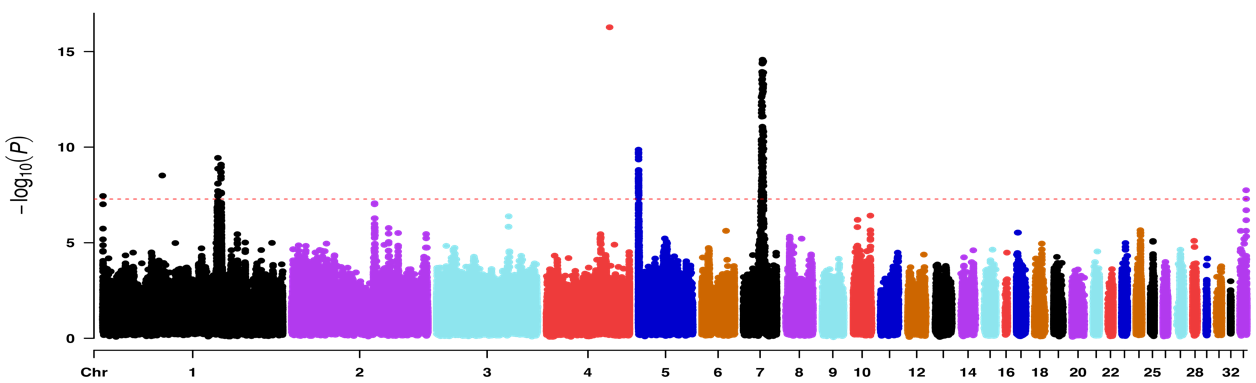
**

**G**

**Other**

**
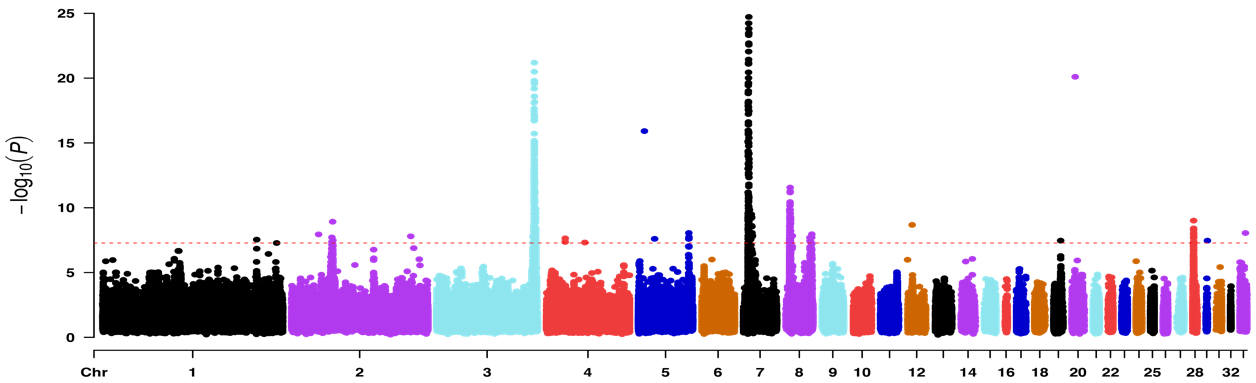
**

**Fig. S4** Manhattan plot showing GWAS signals of another 7 classes of metabolites

**
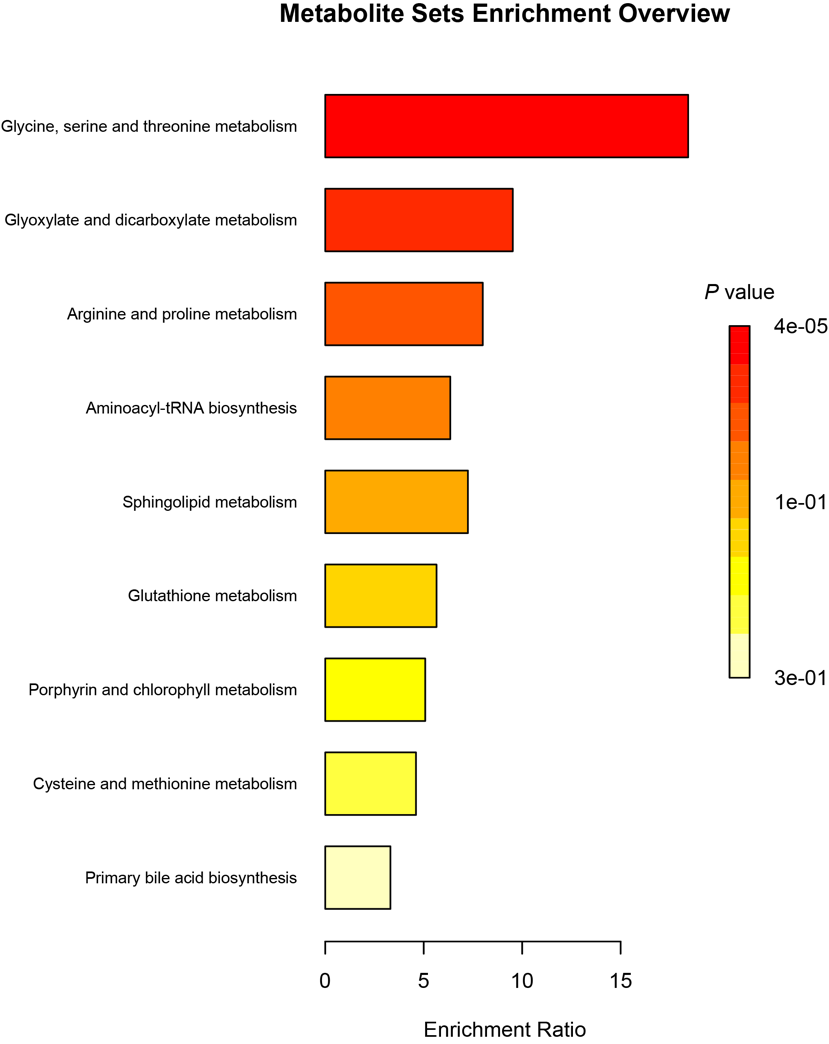
**

**Fig. S5** Analysis of the amino acid metabolites pathway (KEGG) in the metabolites of GWAS signal using MetaboAnalyst

**
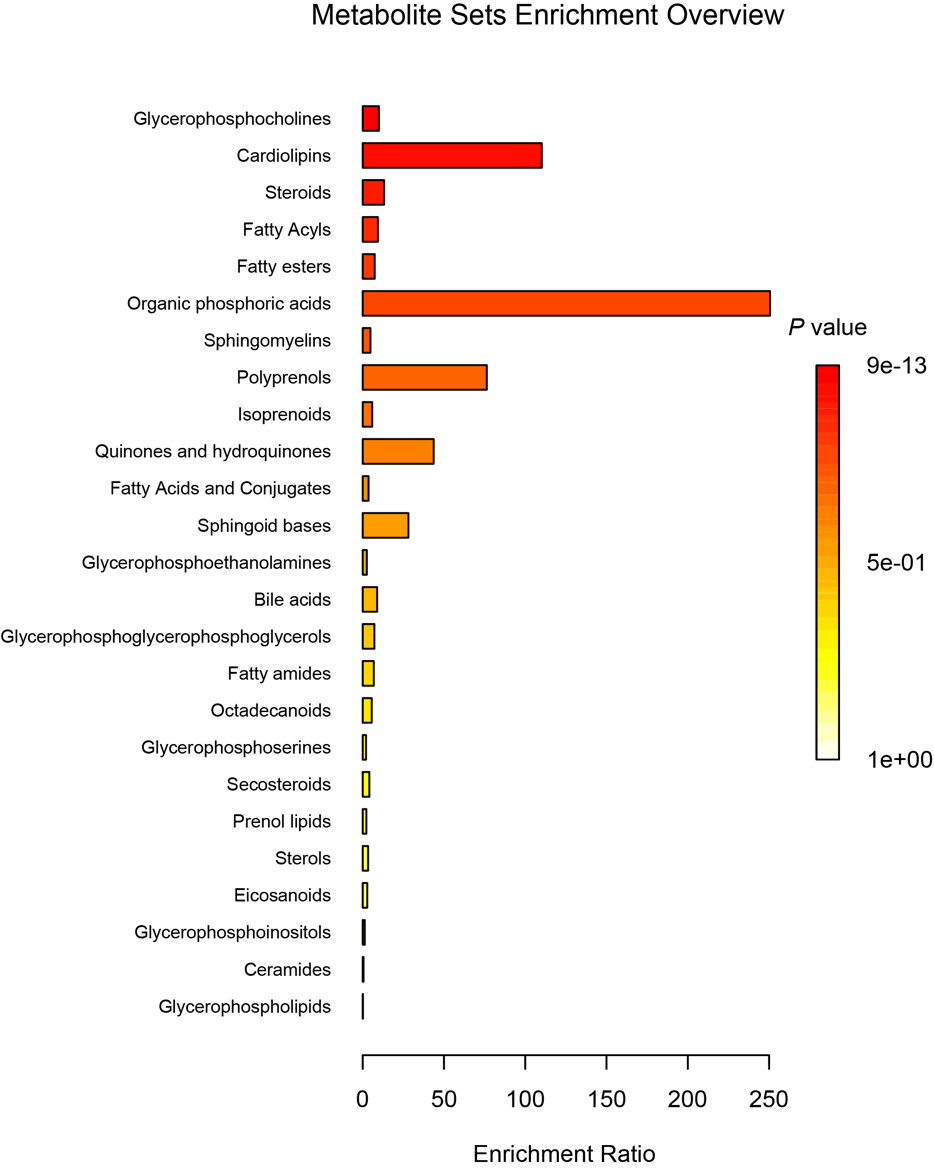
**

**Fig. S6** Classification of lipids in the metabolites of GWAS signal using MetaboAnalyst
